# Survival of cutaneous melanoma based on sex, age, and stage in the United States, 1992–2011

**DOI:** 10.1002/cam4.1152

**Published:** 2017-09-06

**Authors:** Elizabeth Ann L. Enninga, Justin C. Moser, Amy L. Weaver, Svetomir N. Markovic, Jerry D. Brewer, Alexey A. Leontovich, Tina J. Hieken, Lynne Shuster, Lisa A. Kottschade, Ariadna Olariu, Aaron S. Mansfield, Roxana S. Dronca

**Affiliations:** ^1^ Department of Oncology Division of Medical Oncology Mayo Clinic 200 1st Street SW Rochester Minnesota 55905 France; ^2^ Huntsman Cancer Institute Divisions of Hematology and Oncology University of Utah 30 N 1900 E Salt Lake City Utah 84132 France; ^3^ Biomedical Statistics and Informatics Mayo Clinic 200 1st Street SW Rochester Minnesota 55905 France; ^4^ Department of Dermatology Mayo Clinic 200 1st Street SW Rochester Minnesota 55905 France; ^5^ Department of Surgery Mayo Clinic 200 1st Street SW Rochester Minnesota 55905 France; ^6^ Department of Surgery Notre Dame des Aydes Notre Dame des Aydes 11 Rue Franciade Blois 41000 France

**Keywords:** Cutaneous melanoma, female, male, stage, survival

## Abstract

Women diagnosed with cutaneous melanoma have a survival advantage compared to men, which has been hypothesized to be due to difference in behavior and/or biology (sex hormones). It remains controversial whether this advantage is dependent on age or stage of disease. We sought to compare melanoma‐specific survival between females in pre, peri, and postmenopausal age groups to males in the same age group, adjusting for stage of disease. This is a retrospective population‐based cohort study using the Surveillance, Epidemiology, and End Results (SEER) database. Patients diagnosed from 1 January 1992 through 31 January 2011 with primary invasive cutaneous melanoma were included in our cohort. Melanoma‐specific survival was the main outcome studied. Of the 106,511 subjects that were included, 45% were female. Females in all age groups (18–45, 46–54, and ≥55) with localized and regional disease, were less likely to die from melanoma compared to males in the same age group. Among patients with localized and regional disease, the relative risk of death due to melanoma increased with advancing age at diagnosis; this increase was more pronounced among females than males. In contrast, we observed no female survival advantage among patients with distant disease and no effect of age on relative risk of death from melanoma. Females with localized and regional melanoma have a decreased risk of death compared to males within all age groups. Our data show no differences in survival between men and women with metastatic melanoma, indicating that the influence of sex on survival is limited to early stage disease but not confined to pre or perimenopausal age groups.

## Introduction

Malignant melanoma is a cancer of the melanocytes having a 5‐year survival rate of only 15–20% once the tumor has metastasized to distant tissues 1. Over the last few decades the incidence of melanoma has been increasing rapidly in males compared to females of all ages, with the exception of young women (≤39 years) who appear to be at higher risk than young men 2, 3, 4. Yet, women appear to have a survival advantage over men that is not yet understood. Epidemiological studies have confirmed sex as an independent prognostic factor after adjusting for other known predictive markers including age, Breslow thickness, ulceration, histologic subtype, location, and sentinel lymph node positivity 5, 6, 7, 8, 9. Explanations for these disparities have focused on behavioral versus biological differences between men and women. Traditionally, women practice primary (UV protection) and secondary (doctor visits) preventative care more effectively than men 10. However, this does not fully explain why sex remains an independent prognostic factor after adjustment for lifestyle, nor why the female survival advantage has been observed across continents with vast differences in healthcare delivery 11.

In regard to biology, many studies have shown clear differences in pharmacokinetics, responses to infection/inflammation and hormones between the two sexes 12. Significant differences exist in the absorption, distribution, metabolism, and elimination of drugs between sexes; thus, outcomes in clinical (and animal) research should be reported separately by sex 13, 14. Although melanoma is not classically thought of as a hormone responsive tumor, androgen receptors have been detected on melanoma cells, which may explain why this cancer is more aggressive in men 15. Estrogen may also play a role as some reports suggest the female survival advantage is abrogated in postmenopausal females as estrogen levels decline 16, 17. While some investigators have hypothesized that sex influences only local tumor invasion 18, others believe that the effect is limited to lymphatic or hematogenous metastasis 19, 20. However, studies in patients with metastatic disease are limited and have thus far yielded conflicting results 21, 22, 23.

Thus, we elected to investigate the relationship between sex and survival by utilizing the Surveillance, Epidemiology, and End Results (SEER) database. Specifically, our aim was to delineate whether the survival advantage in women is restricted to early stage malignant melanoma, as previously reported, or whether it may persist in advanced or metastatic disease. Herein, we compare the risk of death due to melanoma across all stages of the disease, exploring the possible sex effect using age groups as a proxy for menopausal status in women.

## Methods

### Study data

We identified cases with a diagnosis of melanoma reported to the National Cancer Institute Surveillance, Epidemiology, and End Results (SEER) program between 1 January 1992 and 31 December 2011. SEER*Stat version 8.2.1 software (National Cancer Institute, Bethesda, MD; http://www.seer.cancer.gov/seerstat; accessed 17 June 2015) was used to obtain de‐identified individual‐level data from 13 cancer registries. Cases of melanoma were identified by specifying site “melanoma of the skin,” which included cases with International Classification of Diseases for Oncology, Third Edition (ICD‐0‐3) codes 8720–8723, 8726–8727, 8730, 8740–8746, 8760–8761, 8770–8773, 8780, and 8790. The analysis was limited to patients 18 years or older who had microscopically confirmed invasive cutaneous melanoma.

First primary diagnosis of melanoma was used and patients were excluded if the report was obtained solely from a death certificate or autopsy report, they had a prior history of another cancer, they had in situ melanoma only with no documented invasive disease, they were alive at last follow‐up with 0 months of follow‐up or the cause of death was unknown. Cases were categorized by histologic subtype as superficial spreading, lentigo maligna, acral lentiginous, nodular, not otherwise specified and other. Histologic category “not otherwise specified” and “other” was not collapsed because the survival patterns were different. As the staging system for melanoma was revised in the American Joint Committee on Cancer (AJCC) Cancer staging manual and was implemented into registries in 2003, SEER historic stage was used. These stages include localized (confined to primary organ, the skin), regional (spread to surrounding organs or local lymph nodes), distant (spread to remote organs from primary tumor or to distant lymph nodes), and unknown. Anatomic sites were classified as face/ear (C440–C443), scalp and neck (C444), trunk (C445), extremities (C446–C447), and not otherwise specified/overlapping codes (C448–C449). Scalp and neck melanomas were analyzed separately because they have poorer survival than melanomas of the face, ear and other anatomic sites 24. Information on race and ethnicity was obtained from tumor registrars who reported melanoma to the SEER program. Identification of cases having Hispanic ethnicities was enhanced with the North American Association of Central Cancer Registries Hispanic Identification Algorithm 25. Vital status and cause of death were obtained through 31 December 2012. Patients were categorized into three groups using age as a proxy for menopausal status in women: age 18–45 for premenopausal, 46–54 for perimenopausal, and 55 and older for postmenopausal.

### Statistical analysis

Statistical analyses were performed using the SAS version 9.3 software package and R version 3.0.2. The outcome of interest was death due to melanoma; survival time was calculated from the date of diagnosis to the date of death or last follow‐up. Outcome results were stratified by disease stage. The effect of sex and age at diagnosis on death due to melanoma was evaluated two ways. First, the predicted log hazards were estimated in a Cox proportional hazards model that included sex, age as a continuous variable, and their interaction. The nonlinear effect of age was modeled using a penalized smoothing spline with a pspline basis. Forty‐year‐old females were chosen as the reference in the graphical display of relative risk of death due to melanoma versus age 26. Second, stratified by the three prespecifed age groups, melanoma‐specific survival was estimated separately for males and females using the Kaplan–Meier method and Cox models were fit to evaluate the association between sex and death due to melanoma. These analysis were conducted for the overall cohort at baseline and for cohorts defined on the condition of surviving 1, 3, or 5 years following melanoma diagnosis. Lastly, additional Cox models were fit to evaluate the association between sex and death due to melanoma within strata defined by Breslow depth, histologic subtype, anatomic site, and stage distant disease. Associations were summarized using the hazard ratios (HR) and corresponding 95% confidence intervals (CI) derived from the parameter estimates in the Cox models. The proportional hazards assumption for the Cox models were graphically assessed by plotting the scaled Schoenfeld residuals as a function of rank follow‐up time and the assumption was not violated for sex or age. All calculated *P*‐values were two‐sided and *P*‐values less than 0.05 were considered statistically significant.

## Results

From 1992 to 2011, 201,719 cases of melanoma of the skin were identified in the SEER database. Patient exclusions as defined above were for a diagnosis of melanoma in situ (*n* = 75,634), a history of prior cancer (*n* = 17,331), no pathology confirmation of melanoma (*n* = 519), diagnosis solely from a death certificate or autopsy report (*n* = 12), unknown cause of death was unknown (*n* = 509), and survival time of 0 months or not available (*n* = 1203).

Our final study population consisted of 106,511 patients with invasive cutaneous melanoma (Table 1). Of these patients, 55.2% were male and 44.8% were female. Of the melanoma cases identified in females, the greatest number of cases (44.8%) was identified in the postmenopausal group, followed by the premenopausal (36.0%) and perimenopausal age groups *(*19.2%). The majority of melanoma cases identified in males were seen in the over 55 age group (59.6%). Most patients were Caucasian of non‐Spanish‐Hispanic‐Latino ethnicity having thin melanomas of 0.01–1.00 mm Breslow depth. Superficial spreading was the most common specified histology. In regard to anatomical site, the extremities were the most common location for melanoma (44.1%), followed by the trunk (33.6%), the face and ears (11.8%), and the scalp and neck (6.7%). As expected, males reported more head, neck and trunk melanomas compared with females who had melanoma most often on their extremities.

**Table 1:** Patient demographic and clinical characteristics

Characteristic	Female (*N* = 47,687)	Male (*N* = 58,824)	Total (*N* = 106,511)
Age at diagnosis (years)			
18–45	17,157 (36.0%)	1275 (21.7%)	29,916 (28.1%)
46–54	9156 (19.2%)	11,013 (18.7%)	20,169 (18.9%)
≥55	21,374 (44.8%)	3505 (59.6%)	56,426 (53.0%)
Race, *n* (%)			
American Indian/Alaska native	112 (0.2%)	120 (0.2%)	232 (0.2%)
Asian or Pacific Islander	480 (1.0%)	466 (0.8%)	946 (0.9%)
Black	278 (0.6%)	242 (0.4%)	520 (0.5%)
Unknown	1513 (3.2%)	1586 (2.7%)	3099 (2.9%)
White	45,304 (95.0%)	56,410 (95.9%)	101,714 (95.5%)
Ethnicity among Whites, *n* (%)			
Non‐Spanish‐Hispanic‐Latino	43,370 (95.7%)	55,067 (97.6%)	98,437 (96.8%)
Spanish‐Hispanic‐Latino	1934 (4.3%)	1343 (2.4%)	3277 (3.2%)
Ethnicity, *n* (%)			
Non‐Spanish‐Hispanic‐Latino	45,694 (95.8%)	57,439 (97.6%)	103,133 (96.8%)
Spanish‐Hispanic‐Latino	1993 (4.2%)	1385 (2.4%)	3378 (3.2%)
Stage at diagnosis, *n* (%)			
Distant (stage IV)	1212 (2.5%)	2460 (4.2%)	3672 (3.4%)
Localized (stage I–II)	40,968 (85.9%)	47,900 (81.4%)	88,868 (83.4%)
Regional (stage III)	4054 (8.5%)	6530 (11.1%)	10,584 (9.9%)
Unstaged	1453 (3.0%)	1934 (3.3%)	3387 (3.2%)
Breslow depth, *n* (%)			
No mass/tumor found	367 (0.8%)	790 (1.3%)	1157 (1.1%)
0.01–1.00 mm	31,076 (65.2%)	34,428 (58.5%)	65,504 (61.5%)
1.01–2.00 mm	6061 (12.7%)	8535 (14.5%)	14,596 (13.7%)
2.01–4.00 mm	3149 (6.6%)	4949 (8.4%)	8098 (7.6%)
>4.00 mm	1819 (3.8%)	3193 (5.4%)	5012 (4.7%)
Unknown	5215 (10.9%)	6929 (11.8%)	12,144 (11.4%)
Histologic subtype, *n* (%)			
Superficial spreading	18,466 (38.7%)	19,857 (33.8%)	38,323 (36.0%)
Lentigo maligna	2278 (4.8%)	4577 (7.8%)	6855 (6.4%)
Acral lentigonous	662 (1.4%)	565 (1.0%)	1227 (1.2%)
Nodular	2947 (6.2%)	4622 (7.9%)	7569 (7.1%)
NOS	21,480 (45.0%)	26,516 (45.1%)	47,996 (45.1%)
Others	1854 (3.9%)	2687 (4.6%)	4541 (4.3%)
Anatomic site, *n* (%)			
Face/ears	4146 (8.7%)	8461 (14.4%)	12,607 (11.8%)
Scalp/neck	1833 (3.8%)	5351 (9.1%)	7184 (6.7%)
Trunk	12,319 (25.8%)	23,433 (39.8%)	35,752 (33.6%)
Extremities	27,975 (58.7%)	18,975 (32.3%)	46,950 (44.1%)
NOS/overlapping	1414 (3.0%)	2604 (4.4%)	4018 (3.8%)

### Melanoma‐specific survival

Among the 47,687 females, 3,638 (7.6%) died due to melanoma, 5,787 (12.1%) died due to other causes, and 38,262 (80.2%) were alive at last follow‐up. Among the 58,824 males, 7,411 (7.6%) died due to melanoma, 10,246 (17.4%) died due to other causes, and 41,167 (70.0%) were alive at last follow‐up. The median (IQR, interquartile range) duration of follow‐up after melanoma diagnosis was 8.2 (4.3–13.3) and 7.8 (4.0–12.9) years for females and males, respectively. The median time to death due to melanoma was 2.3 (0.9–4.8) and 2.2 (4.0–0.9–4.3) years for females and males, respectively.

**Figure 1:**
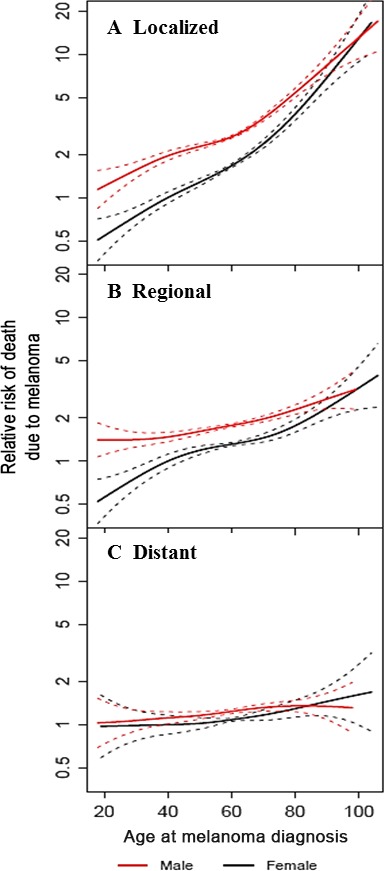
Relative risk of death due to melanoma by sex and age at diagnosis, according to disease stage. In each figure, 40‐year‐old females are the reference (relative risk of 1). (A) localized; (B) regional; (C) distant.

Figure 1 illustrates the effect of sex and age on the risk of death due to melanoma, separately by stage of disease. Age was evaluated as a continuous variable and the nonlinear effect of age was modeled using a penalized smoothing spline. In each figure, 40‐year‐old females were chosen as the referent (relative risk of 1). Among patients with localized and regional disease, the relative risk of death due to melanoma increased with advancing age at diagnosis; however, this increase was more pronounced among females than males. The relative risk of death from melanoma did not vary with age at diagnosis among patients with distant disease.

**Figure 2:**
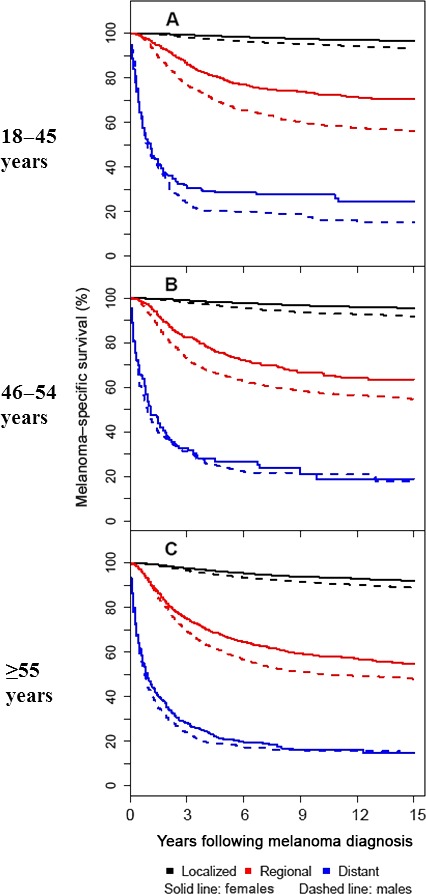
Melanoma‐specific survival according to sex, stage, and age at diagnosis. Each patient's full follow‐up was considered, however, the figures have been truncated at 15 years after diagnosis. (A) 18–45 years at diagnosis; (B) 46–54 years at diagnosis; (C) ≥55 years at diagnosis).

Among patients with localized disease, the risk of death due to melanoma was greater in men than women after adjusting for age (HR 1.59, 95% CI 1.49–1.70). This increased risk in males versus females was greatest in the 18–45 age group (HR 2.05, CI 1.79–2.35) followed by the 46–54 age group (HR 1.89, CI 1.62–2.20) and the ≥55age group (HR 1.42, CI 1.30–1.54) (Table 2, Fig. 2). Among patients with regional disease, the risk of death due to melanoma for males was 1.4 times that of females after adjusting for age (HR 1.37, 95% CI 1.28–1.47). This increased risk was also greatest in the 18–45 age group. In contrast, among patients with distant disease, females were just as likely as males to die of melanoma (HR 1.10, 95% CI 1.01–1.20) after adjusting for age. The analyses were repeated to further adjust for calendar year of diagnosis and given that there were no appreciable differences in the hazard ratios for the sex effect the results adjusted for calendar year have not been presented.

**Table 2:** Association between sex and death due to melanoma, stratified by stage and age at diagnosis

Stage	Age group (years)	No. of deaths due to melanoma/no. of patients		HR (95% CI) males versus females	*P*‐value
		Males	Females		
Localized disease (stage I–II)	18–45	501/10,528	348/15,351	2.05 (1.79, 2.35)	<0.001
	46–54	510/9113	235/8103	1.89 (1.62, 2.20)	<0.001
	≥55	1845/28,259	840/17,514	1.42 (1.30, 1.54)	<0.001
	All ages, age adjusted^a^	2856/47,900	1423/40,968	1.59 (1.49, 1.70)	<0.001
Regional disease (stage III)	18–45	500/1400	255/1057	1.65 (1.42, 1.92)	<0.001
	46–54	430/1144	189/647	1.40 (1.18, 1.66)	<0.001
	≥55	1514/3986	751/2350	1.25 (1.14, 1.36)	<0.001
	All ages, age adjusted^a^	2444/6530	1195/4054	1.37 (1.28, 1.47)	<0.001
Distant disease (stage IV)	18–45	298/411	148/223	1.14 (0.93, 1.38)	0.21
	46–54	303/446	108/164	1.07 (0.86, 1.33)	0.57
	≥55	1105/1603	535/825	1.09 (0.99, 1.21)	0.09
	All ages, age adjusted^a^	1706/2460	791/1212	1.10 (1.01, 1.20)	0.028

HR, hazard ratio; CI, confidence interval.

a Age as a continuous variable, not categorized into three age groups, was included in the Cox proportional hazards models.

**Figure 3:**
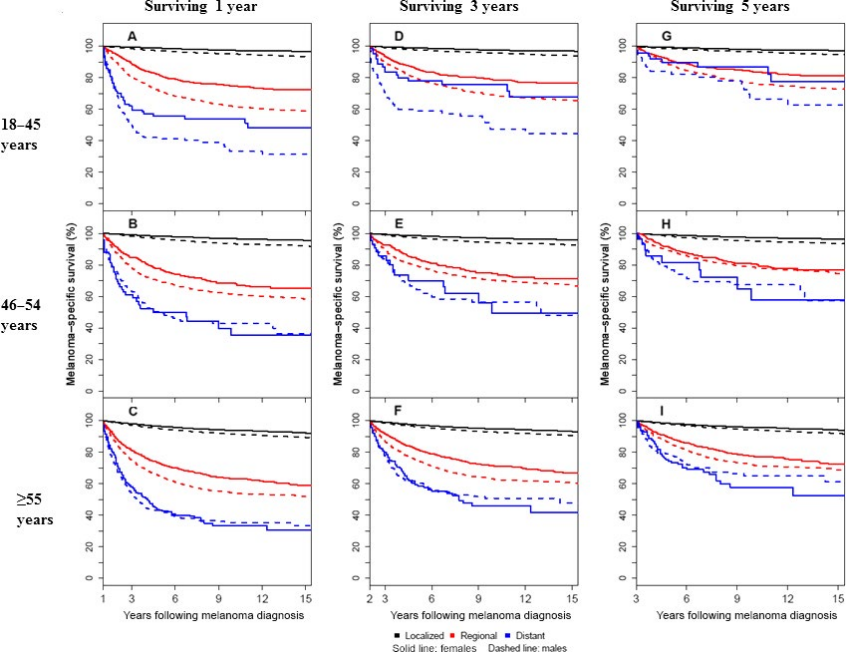
Conditional melanoma‐specific survival according to sex, stage, and age at diagnosis. Each patient's full follow‐up was considered, however, the figures have been truncated at 15 years after diagnosis. (A) 18–45 years at diagnosis and surviving 1 year (*n* = 25,508 localized, *n* = 2342 regional, and *n* = 279 distant); (B) 46–54 years at diagnosis and surviving 1 year (*n* = 16,970 localized, *n* = 1679 regional, and *n* = 265 distant); (C) ≥55 years at diagnosis and surviving 1 year (*n* = 44,209 localized, *n* = 5555 regional, and *n* = 883 distant); (D) 18–45 years at diagnosis and surviving 3 years (*n* = 22,613 localized, *n* = 1754 regional, and *n* = 122 distant); (E) 46–54 years at diagnosis and surviving 3 years (*n* = 14,491 localized, *n* = 1188 regional, and *n* = 124 distant); (F) ≥55 years at diagnosis and surviving 3 years (*n* = 34,741 localized, *n* = 3316 regional, and *n* = 353 distant); (G) 18–45 years at diagnosis and surviving 5 years (*n* = 19,641 localized, *n* = 1376 regional, and *n* = 87 distant); (H) 46–54 years at diagnosis and surviving 5 years (*n* = 12,131 localized, *n* = 931 regional, and *n* = 79 distant); (I) ≥55 years at diagnosis and surviving 5 years (*n* = 26,625 localized, *n* = 2301 regional, and *n* = 200 distant).

Comparison between males and females of melanoma‐specific survival conditional on surviving 1, 3, or 5 years following diagnosis are summarized in Table 3 and Figure 3. The survival advantage for female patients with localized disease persisted for all 3 age groups even among patients who survived 5 years after diagnosis. This was also observed for patients with regional disease diagnosed between 18 and 45 years of age. For patients with regional disease diagnosed between 46 and 54 years of age, patient sex no longer significantly differentiated survival after 2 years of conditional survival from melanoma (HR 1.22, 95% CI 0.98–1.52, figure not shown). For patients with regional disease diagnosed at 55 years of age and older, patient sex no longer significantly differentiated survival after 5 years of conditional survival from melanoma. (HR 1.17, 95% CI 0.93–1.47).

**Table 3:** Association between sex and death due to melanoma, stratified by stage and age at diagnosis, conditional on surviving 1, 3, or 5 years, respectively

Stage	Age group (years)	Death due to melanoma conditional on surviving 1 year		Death due to melanoma conditional on surviving 3 years		Death due to melanoma conditional on surviving 5 years	
		HR (95% CI) males versus females	*P*‐value	HR (95% CI) males versus females	*P*‐value	HR (95% CI) males versus females	*P*‐value
Localized disease (stage I–II)	18–45	2.03 (1.77, 2.33)	<0.001	1.85 (1.58, 2.17)	<0.001	1.69 (1.39, 2.05)	<0.001
	46–54	1.90 (1.63, 2.23)	<0.001	1.83 (1.52, 2.20)	<0.001	1.82 (1.45, 2.29)	<0.001
	≥55	1.42 (1.30, 1.54)	<0.001	1.40 (1.25, 1.56)	<0.001	1.42 (1.23, 1.63)	<0.001
	All ages, age adjusted^a^	1.60 (1.50, 1.71)	<0.001	1.57 (1.45, 1.70)	<0.001	1.55 (1.40, 1.72)	<0.001
Regional disease (stage III)	18–45	1.64 (1.40, 1.93)	<0.001	1.46 (1.16, 1.83)	0.001	1.61 (1.16, 2.23)	0.004
	46–54	1.30 (1.08, 1.56)	0.005	1.11 (0.85, 1.46)	0.44	0.94 (0.65, 1.38)	0.76
	≥55	1.34 (1.21, 1.48)	<0.001	1.27 (1.09, 1.49)	0.003	1.17 (0.93, 1.47)	0.19
	All ages, age adjusted^a^	1.41 (1.31, 1.52)	<0.001	1.30 (1.15, 1.46)	<0.001	1.23 (1.04, 1.46)	0.017
Distant disease (stage IV)	18–45	1.41 (0.99, 2.01)	0.06	1.67 (0.75, 3.71)	0.20	1.76 (0.52, 5.88)	0.36
	46–54	0.94 (0.64, 1.38)	0.75	1.03 (0.49, 2.19)	0.93	0.85 (0.26, 2.76)	0.78
	≥55	1.07 (0.88, 1.30)	0.50	0.88 (0.59, 1.30)	0.51	0.82 (0.39, 1.70)	0.59
	All ages, age adjusted^a^	1.10 (0.95, 1.29)	0.21	1.02 (0.75, 1.41)	0.88	0.97 (0.56, 1.68)	0.92

HR, hazard ratio; CI, confidence interval.

a Age as a continuous variable, not categorized into three age groups, was included in the Cox proportional hazards models.

Among patients with localized disease, the risk of death due to melanoma was consistently greater in males than females regardless of Breslow depth, histologic subtype, or anatomical location (Table 4). Among patients with regional disease, the risk of death due to melanoma was consistently greater in males than females regardless of the Breslow depth or histologic subtype, except the association was attenuated among the small subset with lentigo maligna. In addition, the risk of death due to melanoma was consistently greater in males than females with melanoma on the face/ears, trunk, or extremities, but the risk was not differentially elevated among those with melanoma on the scalp or neck.

**Table 4:** Among patients with localized (stage I–II) and regional disease (stage III), separately, association between sex and death due to melanoma, stratified by melanoma characteristics

Characteristic	Among patients with localized disease			Among patients with regional disease		
	No. of deaths due to melanoma/no. of patients	HR (95% CI) males versus females	*P*‐value	No. of deaths due to melanoma/no. of patients	HR (95% CI) males versus females	*P*‐value
Breslow depth (mm), *n* (%)						
0.01–0.99 mm	1255/63,285	1.76 (1.57, 1.98)	<0.001	375/1540	1.65 (1.32, 2.05)	<0.001
1.00–2.00 mm	1179/12,187	1.57 (1.39, 1.78)	<0.001	587/2158	1.42 (1.20, 1.68)	<0.001
2.01–4.00 mm	1023/5194	1.46 (1.28, 1.66)	<0.001	936/2662	1.39 (1.21, 1.59)	<0.001
>4.00 mm	436/1780	1.59 (1.29, 1.96)	<0.001	1179/2833	1.35 (1.19, 1.52)	<0.001
Unknown	386/9421	1.55 (1.26, 1.90)	<0.001	456/1101	1.16 (0.96, 1.41)	0.13
Histologic subtype, *n* (%)						
Superficial spreading	1259/35,694	1.87 (1.66, 2.10)	<0.001	602/2063	1.73 (1.45, 2.06)	<0.001
Lentigo maligna	173/6508	2.26 (1.54, 3.32)	<0.001	48/175	1.22 (0.65, 2.28)	0.53
Acral lentigonous	89/784	1.50 (0.99, 2.28)	0.06	147/364	1.46 (1.05, 2.04)	0.024
Nodular	865/4559	1.72 (1.48, 1.98)	<0.001	1051/2634	1.40 (1.23, 1.59)	<0.001
NOS	1694/38,169	1.70 (1.54, 1.88)	<0.001	1525/4343	1.25 (1.12, 1.39)	<0.001
Others	199/3154	1.83 (1.35, 2.48)	<0.001	266/1005	1.34 (1.04, 1.73)	0.026
Anatomic site, *n* (%)						
Face/ears	591/10,833	1.69 (1.40, 2.04)	<0.001	318/1152	1.47 (1.14, 1.90)	0.004
Scalp/neck	517/5629	1.38 (1.12, 1.70)	0.003	459/1134	1.09 (0.88, 1.35)	0.45
Trunk	1588/31,276	1.55 (1.39, 1.73)	<0.001	1160/3101	1.39 (1.21, 1.59)	<0.001
Extremities	1560/40,687	1.77 (1.60, 1.96)	<0.001	1456/4556	1.41 (1.27, 1.56)	<0.001
NOS/overlapping	23/443	1.67 (0.69, 4.07)	0.26	246/641	0.95 (0.73, 1.23)	0.68

HR, hazard ratio; CI, confidence interval.

The site of distant disease was available in SEER for 1651 of the 1813 patients with melanoma who presented with stage IV disease diagnosed from 2004 to 2011. Table 5 summarizes the association between sex and death due to melanoma stratified by stage.

**Table 5:** Association between sex and death due to melanoma, stratified by site of distant disease among patients diagnosed with distant disease (stage IV) in 2004–2011

Site of distant disease	No. of deaths due to melanoma/no. of patients		HR (95% CI) males versus females	*P*‐value
	Males	Females		
M1a	78/144	35/78	1.37 (0.92, 2.04)	0.12
M1b	89/133	43/71	1.11 (0.77, 1.60)	0.56
M1c	605/845	251/371	1.02 (0.88, 1.18)	0.77
Unknown	40/101	22/61	–	–

HR, hazard ratio; CI, confidence interval.

## Discussion

Many studies have found a female survival advantage among patients with melanoma which is independent of differences in detection or diagnosis. As no biological rationale for this advantage has been fully identified, we analyzed melanoma‐specific survival between males and females for different stages of disease and different age groups to explore whether there might be an association with hormonal status. Although previous studies using the SEER database have suggested a female survival benefit in patients with melanoma of the head and neck and in non‐Hispanic patients under the age of 40, neither of these studies analyzed survival in regards to age and tumor stage as was done in this study 8, 27. Our results confirmed the female survival benefit among patients treated for early stage melanoma, namely localized (stage I and II) and regional (stage III) disease; however, not for patients with distant (stage IV) disease. Our results seem contradictory from a recently reported analysis of three large European clinical trials showing that females with distant disease maintained a survival benefit compared to male patients 7. While both our study and the European study looked at patients over similar time periods, we used the SEER database and included patients who received any treatment for distant disease, whereas the previous study looked only at patients who received one of four possible chemotherapies, all of which included an alkylating agent such as dacarbazine or temozolomide. Their results may have been confounded by the fact that women respond better to both dacarbazine and temozolomide compared to men 28, 29. Similar to our findings, the female survival advantage seemed to decrease in patients with higher metastatic tumor burden 7. Studies using other large registries, which included therapies other than alkylating agents, similarly did not detect a female survival benefit for patients with distant disease 21, 23. Clearly there is a need to clarify the biology behind these contradictory conclusions.

It's been suggested by some that females continue to have superior survival with increasing age compared to males following both localized and regional melanoma diagnoses 7, 23, 30. Conversely, others have shown no survival difference between male and female melanoma patients with increasing age, mainly due to an increasing risk of death with age in females 6, 16. Traditionally, women of all age groups tend to visit their health care providers more frequently, avoid excessive sun exposure and complete skin checks compared to men 10. Yet, these behavioral differences between men and women cannot fully account for the observed differences in cancer survival. Although the survival advantage for females decreased with the advancing age group at diagnosis, among the patients with localized disease in the ≥55 age group we found men were still 1.4 times more likely than women to die due to melanoma. Unsurprisingly, both males and females had increasing risk of death due to melanoma with increasing age at diagnosis. However, this increase was steeper for women than for men, suggesting that age alone is not the only contributing factor.

Although our study suggests a potential association of estrogen with melanoma based on the finding of improved survival in women with local and regional disease, many epidemiologic reports suggest that estrogens do not affect melanoma citing studies of oral contraceptives, hormone replacement therapy and pregnancy 31. Conversely, others have found estrogen receptor (ER)β is expressed on metastatic melanoma tumors and loss of expression results in increased tumor invasion 32. ERβ expression has also been shown to decline after menopause, which may explain the loss of the female survival advantage in women over 55 33. Clearly collecting specific information on menopausal status and exogenous hormone use (inclusive of contraception, menopausal therapy, and antiestrogen therapy used as part of treatment and prevention of other hormone‐sensitive neoplasms) will be essential for future research on the effects of sex hormones on the incidence, prognosis, and survival of patients diagnosed with melanoma. As estrogen could have a protective role due to the observed better outcome in females with melanoma, we analyzed survival using age as a proxy for menopausal status and observed a survival advantage for women across all age groups compared to men.

While a female survival benefit was seen for patients with localized and regional disease in all age categories, we did not observe similar differences in patients with distant disease and higher tumor burden. One postulated reason could be the drastically different immunologic changes associated with metastatic melanoma compared to earlier stages of disease. For instance, systemic immunity is biased toward a state of chronic inflammation in patients with distant disease that is not seen in patients with early stage melanoma or healthy volunteers 34. Aging is also associated with increased markers of chronic inflammation which may play a role in the decreased survival of older patients with all stages of melanoma 35. New therapies that harness the immune system to destroy the tumor have had great success in melanoma, indicating the importance of host immunity for improved survival 36. Since immune checkpoint agents have only recently been FDA approved for melanoma, it is unlikely that patients taking immune checkpoint inhibitors are represented in this study. With more patients being treated with these therapies, it will be important to look for sex differences in survival and side effect profiles in this population.

We are limited in that our dataset only allowed us to evaluate age and not actual menopausal status to address the role of menopause in mediating melanoma survival. Additionally, hormone use, patient comorbidities, treatment preferences and other possible factors that influence survival were unavailable with the SEER database. Due to the fact that the AJCC staging system was only implemented into registries in 2003, we elected to use the SEER historic stage for all patients. We performed additional analyses regarding the association between sex and death due to melanoma for localized and regional disease patients stratified by melanoma characteristics such as Breslow depth, histopathological subtype, and anatomic site. These limitations should be weighed against the strengths of the SEER including population‐based data, referral bias elimination, detailed tumor information and large sample size to adequately power analyses. Nevertheless, we believe that this study provides a rationale for further studies investigating hormonal‐ and sex‐based differences in the outcome of malignant melanoma and the effect of sex on response to melanoma therapies.

In summary, this study utilizes the SEER database to study the female survival advantage not only in localized and regional melanoma, but also in patients presenting with distant disease. The large number of patients allowed us to more clearly describe the epidemiologic pattern of melanoma‐specific survival in the United States in men and women across three different age groups. Data from our study indicate that, compared to males, females have improved survival for localized and regional disease, but that this sex difference is lost among patients presenting with distant disease. Further study of the biological and environmental differences between male and female patients is warranted. Understanding the basis for these differences has the potential to improve outcomes for cutaneous melanoma patients.

## Conflict of Interest

None declared.
